# Unhealthy work allowance and profile of workers at a federal university before and after adoption of the concession module of Integrated Health Care Subsystem for Civil Servants

**DOI:** 10.47626/1679-4435-2024-1333

**Published:** 2025-09-22

**Authors:** Cristiane de Almeida Pereira, Eliane Marina Palhares Guimarães, Mery Natali Silva Abreu

**Affiliations:** 1 Postgraduate Program in Health Services Management, Federal University of Minas Gerais, Belo Horizonte, MG, Brazil

**Keywords:** computer systems, information systems, occupational health, occupational risks., sistemas de computação, sistemas de informação, saúde do trabalhador, riscos ocupacionais.

## Abstract

**Introduction:**

In 2018, the Integrated Health Care Subsystem for Civil Servants Concession Module for Occupational Allowances (Módulo de Concessão para Adicionais Ocupacionais do Subsistema Integrado de Saúde do Servidor) was established as a pilot project. This computerized system was created to enhance the processes for granting occupational allowances, ensure compliance with current legislation, and record this information within the federal public service.

**Objectives:**

To identify the positions, sectors, and risk units at Federal University of Minas Gerais, to characterize the profile of employees engaged in unhealthy activities, and to quantify the number of employees who received the allowance before and after the system’s implementation.

**Methods:**

A quantitative design with descriptive and analytical aims, utilizing secondary data obtained from payroll control spreadsheets.

**Results:**

The data indicated that the institution’s typical employee is female, aged between 40 and 60 years, holds an undergraduate degree, occupies health related positions, and works in a hospital. Moreover, there was a 26.8% reduction in the number of employees receiving the allowance, a 30.0% reduction in the number of units, and a 64.7% reduction in the number of organizational units with recognized compensable unhealthy conditions.

**Conclusions:**

Although this remote management system standardized procedures, allowed remote access to information, and reduced expenses related to unhealthy allowances, it was not developed with a focus on disease or accident prevention, nor on the promotion of worker health.

## Abstract (pt)

**Introdução:**

Em 2018, instituiu-se o Módulo de Concessão para Adicionais Ocupacionais do Subsistema Integrado de Saúde do Servidor, em forma de projeto piloto. Esse sistema informatizado foi criado para aprimorar os processos de concessão de adicionais ocupacionais, garantir a conformidade com a legislação vigente e fazer o registro dessas informações no serviço público federal.

**Objetivos:**

Apontar os cargos, setores e unidades de risco na Universidade Federal de Minas Gerais, identificar o perfil dos servidores que trabalham em atividades insalubres e quantificar o número de servidores que receberam o adicional antes e após a adoção do sistema.

**Métodos:**

Abordagem quantitativa, com finalidade descritiva e analítica, utilizando dados secundários provenientes de planilhas de controle de pagamento.

**Resultados:**

Os dados apontaram que o perfil do servidor da instituição é mulher, com idade entre 40 e 60 anos, formação superior completa, exercendo cargos da área de saúde e trabalhando em ambiente hospitalar. Também demonstraram que houve uma redução de 26,8% na quantidade de servidores, de 30,0% nas unidades e de 64,7% nas unidades organizacionais com reconhecimento de insalubridade indenizável.

**Conclusões:**

Esse sistema de telegestão, apesar de padronizar, permitir o acesso remoto às informações e resultar em redução de gastos com o pagamento do adicional de insalubridade, não foi desenvolvido com foco em ações para a prevenção de adoecimento e de acidentes, nem para a promoção da saúde do trabalhador.

## INTRODUCTION

Worker Health Surveillance (Vigilância em Saúde do Trabalhador, VISAT), instituted by Ordinance No. 3.120 of 1998 of the Ministry of Health of Brazil, comprises the set of actions aimed at detecting, understanding, researching, and analyzing the determinant and conditioning factors of health hazards related to work processes and environments. VISAT encompasses social, technological, organizational, and epidemiological dimensions, with the purpose of planning, executing, and evaluating interventions on these risk factors for occupational illness, seeking their eradication and control.

Environmental assessments for diagnosing risk factors and hazardous situations are incorporated into the activities of VISAT. In this context, characterizing what is unhealthy in the work environment is one of the tasks undertaken by professionals in this field. Unhealthy conditions characterize factors that can lead to illness. In the workplace, they apply to certain activities and operations in accordance with Article 189 of Decree No. 5,452 of 1943, which instituted the Consolidation of Labor Laws (Consolidação das Leis do Trabalho, CLT):

Art.189 - Activities or operations shall be considered unhealthy when their nature, conditions, or methods of work expose employees to agents harmful to health above the tolerance limits established on account of the nature and intensity of the agent and the duration of exposure to its effects.^1^

This definition was specified by Regulatory Standard (Norma Regulamentadora, NR) No. 15 of 1977:

15.1 Activities or operations are considered unhealthy when they take place:
15.1.1 Above the tolerance limits provided in Annexes No. 1, 2, 3, 5, 11, and 12;
15.1.2 *(Repealed by Ministry of Labor and Employment Ordinance No. 3.751 of November 23, 1990)*
15.1.3 In the activities mentioned in Annexes No. 6, 13, and 14
15.1.4 Demonstrated by a workplace inspection report, as set forth in Annexes No. 7, 8, 9, and 10.^2^

In Brazil, performing work under unhealthy conditions entitles the worker to receive an unhealthy work allowance. To qualify for this compensation, an expert report is required. The characterization and classification of both unhealthy and hazard conditions are carried out through a technical assessment conducted by an occupational physician, or an occupational safety engineer registered with the Ministry of Labor.^1^

Oliveira^3^ reflects that this monetary supplement is the least acceptable means of protecting workers from occupational hazards, since the amount paid is more advantageous to the employer than the costs required to improve the work environment. This situation warrants rigorous studies for the collective construction of knowledge and strategies aimed at making work environments safer and healthier.

From this perspective, the present study aimed to investigate the working conditions of the technical administrative staff in education (TAE) at Federal University of Minas Gerais (UFMG).

Until August 2018, an institutional occupational physician or occupational safety engineer conducted visits to each staff member’s workstation to assess work activities and the environment. Following the expert evaluation, an individualized report was prepared for that employee. If another staff member in the same department and position initiated a separate procedure, the expert was required to perform a new assessment and draft a new report. Therefore, until that date, reports were fully personalized. From September 2018, expert reports began to be produced on an environmental basis - that is, by sector and job position - covering more than one individual per report. In addition to this change, the reports started to be entered in a standardized document within one of the modules that compose the Integrated Personnel Administration System (Sistema Integrado de Administração de Pessoal, SIAPE) of the Federal Public Administration, and to be included in the employee’s personal file. SIAPE is responsible for the payment of federal civil servants.

The Integrated Health Care Subsystem for Civil Servants (Subsistema Integrado de Saúde do Servidor, SIASS) Concession Module for Occupational Allowances, which belongs to SIAPE, was developed in 2018 by the former Ministry of Planning, Development and Management. Its objective was to improve the processes for granting occupational allowances in federal bodies, ensure compliance with current legislation, and record this information within the federal public service. The module was released as a pilot project, with the participation of the National Social Security Institute (Instituto Nacional do Seguro Social), Oswaldo Cruz Foundation, Ministry of Health, National Cancer Institute, UFMG, Federal University of Rio Grande do Norte, and Federal University of Rio de Janeiro.

The present study’s lead researcher, an occupational physician at the UFMG, participated in implementing this computerized system. Motivated by a desire to deepen knowledge on the topic, the study aimed to improve worker health measures and streamline occupational risk compensation management. Its objectives were to list institutional positions, sectors and risk areas; characterize employees in unhealthy roles; and quantify allowance recipients before and after system adoption.

## METHODS

This study used a quantitative approach and conducted a descriptive analysis using secondary data from two spreadsheets supplied by the Department of Worker Health Care (Departamento de Atenção à Saúde do Trabalhador, DAST), which is the unit responsible for environmental assessments and for issuing expert reports to pay unhealthy work allowances to UFMG staff. These documents list employees and details like job position, sex, age, education level, workplace and salary allowance type. The first spreadsheet covers July 2018, before implementing the SIASS module. The second covers January 2021, after system adoption.

Inclusion criteria: holding a position within institution’s TAE and receiving an unhealthy work allowance for any hazard (biological, chemical or physical). The study focused on these employees because the institution’s central administration chose them as pioneers in the allowance review, they represented the largest staff segment, and the environmental assessment covered all employees in the same role within a given work unit.

Exclusion criteria: staff in the teaching career and recipients of hazard pay or X ray bonus. We excluded professors because their research activities and practical courses require individual evaluations and advance scheduling. Thus, the time required to complete expert reports could exceed what was needed to complete the study. Regarding the exclusion of employees receiving hazard pay, this reflected our focus on mapping unhealthy conditions, a concept distinct from hazard pay. Hazard constitutes a threat to a worker’s life and integrity, meaning it represents the risk of a workplace accident, which can occur even in healthful environments. The X ray bonus was also excluded from this evaluation since it is a salary supplement granted to few employees performing highly specific tasks, and legislation requires special conditions for their duties, such as reduced working hours and appropriate infrastructure and environment.

We compared the spreadsheets listing employees receiving allowances to determine changes in allowance concessions following the introduction of this new SIASS module.

We performed frequency distribution analyses, reporting absolute and relative frequencies, and produced charts. We also applied Pearson’s chi-square test and Student’s *t*-test to compare results before and after system implementation, using a 5% significance level. All analyses were conducted with SPSS version 19.0.

This project received approval from the institution and the UFMG’s Research Ethics Committee, protocol number 4.636.215.

## RESULTS

Data extracted from the spreadsheets managing unhealthy work allowance payments for 2018 and 2021 were organized into tables and charts according to employee profiles. The information covered characteristics such as sex, age, position, education level and work location at UFMG.

In July 2018, UFMG had 8,364 employees; 4,298 held positions within TAE. Of these, 1,289 received unhealthy work allowance, representing 15.4 % of the total workforce and 29.9% of TAE. In January 2021, the university counted 8,115 employees, including 4,163 TAE; 943 received unhealthy work allowance, corresponding to 11.6% of the total workforce and 22.6% of TAE. From 2018 to 2021, there was a 3.79 percentage-point decrease in total staff and a 7.34 percentage-point decrease among TAE. These differences reached statistical significance (p < 0.01).

This study analyzed data from 1,289 employees who received unhealthy work allowance in 2018, and 943 in 2021. This information made it possible to profile the workers and identify the units to which they are assigned within the institution, enabling the mapping of occupational risks at the university under study.

Table 1 shows sociodemographic characteristics for employees who received unhealthy work allowance in 2018 and 2021 at UFMG, in absolute numbers and percentages. Regarding sex, women were the majority in both periods, accounting for over 70% of these employees.

**Table 1:** Sociodemographic characteristics of employees receiving unhealthy work allowance at UFMG in 2018 and 2021

Characteristic	2018 (n = 1,289)		2021 (n = 943)		p-value^*^
	n	%	n	%	
Sex					0.595
Female	937	72.7	695	73.7	
Male	352	27.3	248	26.3	
Education level					< 0.001
Primary education	66	5.1	23	2.4	
Secondary/technical	442	34.3	184	19.5	
Undergraduate	645	50.0	566	60.0	
Postgraduate	136	10.6	170	18.0	
Age range (years)					0.259
20-39	258	20.0	178	18.9	
40-59	895	69.4	682	72.3	
60 and older	136	10.6	83	8.8	
Job category					< 0.001
1 Nursing	703	54.5	572	60.7	
2 Physicians	188	14.6	158	16.8	
3 Laboratory technicians and specialized assistants	217	16.8	112	11.9	
4 Other health professionals	55	4.3	55	5.8	
6 Other health technicians	46	3.6	19	2.0	
5 Health support staff	39	3.0	14	1.5	
7 Maintenance staff	24	1.9	3.0	0.3	
8 Other categories	17	1.3	10	1.1	
Unit					< 0.001
Hospital das Clínicas of UFMG	1,069	82.9	810	85.9	
Department of Worker Health Care	19	1.5	29	3.1	
Institute of Biological Sciences	63	4.9	26	2.8	
Veterinary School	29	2.2	35	3.7	
Other units	109	8.5	43	4.6	

UFMG = Federal University of Minas Gerais; DAST = Departamento de Atenção à Saúde do Trabalhador (Department of Worker Health Care).

* Chi-square test.

Undergraduate degrees were the most common qualification among TAE in both periods. Comparing 2018 and 2021, the share of allowance recipients with an undergraduate degree rose by 10 percentage points. Lower education levels now represent a smaller share: staff with primary education fell by 50% and those with secondary/technical education by 15%. Postgraduate staff, however, increased by nearly 8 percentage points, as shown in Table 1.

The 40-59 age range was most prevalent in both 2018 and 2021, representing about 70% of staff. It rose by 2.9 percentage points over that period. The younger group (20-39 years) then saw a 1.1 percentage-point drop in 2021. Meanwhile, the oldest group (60 years and older) remained the smallest and fell by 1.8 percentage points in 2021 (Table 1).

Health-related job categories received the highest number of unhealthy work allowances in both 2018 and 2021. Nursing saw a 6.2 percentage-point increase in allowance recipients in 2021, making it the top category. Physicians followed with a 2.2 percentage-point rise (Table 1). This trend reflects demand for more professionals, driven by expanded vacancies or expected retirements in these roles.

Among the organizational units (OUs) with recognized unhealthy activities, the Hospital das Clínicas of UFMG (HCUFMG) accounts for over 82% of employees exposed to occupational hazards in both 2018 and 2021. That share increased by 3 percentage points between those years. DAST was the OU with the largest relative increase, doubling the share of workers with compensable occupational risk (Table 1). This jump resulted from a change in entitlement recognition by the expert who conducted the latest evaluation of the OU housing the medical examiners. Previous reports did not deem those activities unhealthy.

The School of Veterinary Medicine saw a 40.5% increase in employees receiving unhealthy work allowance. All other OUs saw a decrease in allowance recipients. At the Institute of Biological Sciences the drop was 43.0% and at the remaining OUs it was about 46.0%, falling from 8.5 to 4.6% of all employees in this study (Table 1).

Regarding other professional categories, Table 2 shows they represent a small share of roles with illness risk, now totaling just over 1%. Some categories no longer include any allowance recipients since 2021, such as accounting technicians and agricultural machinery operators.

**Table 2:** Descending order of other job categories at UFMG with the highest number of unhealthy work allowance recipients

Job category and title	2018		2021	
	n	%	n	%
8 Animal care professionals	11	0.9	10	1.1
9 Graphic arts professionals	2	0.2	0	0.0
10 Graduates from other fields	1	0.1	0	0.0
11 Agricultural machinery operators	1	0.1	0	0.0
12 Accounting technicians	1	0.1	0	0.0
Total	16	1.4	10	1.1

UFMG = Federal University of Minas Gerais.

Table 3 confirms the remaining units account for just 4.4% of TAE in the 2021 sample. Their number fell by 48.8% between 2018 and 2021. In some units, no workers received unhealthy work allowance. These include the School of Pharmacy, the Department of Infrastructure Maintenance and Operations, the Microscopy Center, the School of Fine Arts, the Museum of Natural History and Botanical Garden and the School of Law. In other areas such as the Department of Supply Logistics and Operational Services, the University Sports Center and the School of Physical Education, Physiotherapy and Occupational Therapy, rates stayed the same. Only at Institute of Exact Sciences did they rise slightly.

**Table 3:** Other UFMG units with the highest number and greatest percentage change of employees receiving unhealthy work allowance

Unit	2018		2021		p-value^*^
	n	%	n	%	
School of Dentistry	27	2.1	21	2.2	
School of Pharmacy	21	1.6	0	0.0	
Institute of Exact Sciences	9	0.7	8	0.8	
School of Medicine	11	0.9	6	0.6	
School of Engineering	11	0.9	1	0.1	
Institute of Agricultural Sciences	8	0.6	1	0.1	
Department of Supply Logistics and Operational Services	3	0.2	2	0.2	
Department of Infrastructure Maintenance and Operations	4	0.3	0	0.0	
Institute of Geosciences	3	0.2	1	0.1	< 0.001
Technical College	2	0.2	1	0.1	
Microscopy Center	3	0.2	0	0.0	
University Sports Center	1	0.1	1	0.1	
School of Fine Arts	2	0.2	0	0.0	
School of Physical Education, Physiotherapy and Occupational Therapy	1	0.1	1	0.1	
Museum of Natural History and Botanical Garden	2	0.2	0	0.0	
Infrastructure Coordination	1	0.1	0	0.0	
Total	109	8.6	43	4.4	

UFMG = Federal University of Minas Gerais.

Regarding TAE who lost and those who began receiving the unhealthy work allowance (Table 4), sex showed no significant difference (p = 0.752). Women accounted for over 70% of both groups. HCUFMG employs the majority of at risk staff, confirming earlier findings that women dominate health roles.

**Table 4:** Sociodemographic characteristics of UFMG employees who lost vs. gained the unhealthy work allowance in 2018 and 2021

Characteristic	Lost (n = 453)		Gained (n = 107)		
	n	%	n	%	p-value^*^
Sex					0.752
Female	319	70.4	77	72.0	
Male	134	29.6	30	28.0	
Education level					< 0.001
Primary education	42	9.3	4	3.7	
Secondary/technical	159	35.1	17	15.9	
Undergraduate	192	42.4	67	62.6	
Postgraduate	60	13.2	19	17.8	
Age range (years)					< 0.001
20-39	71	15.7	45	42.1	
40-59	285	62.9	57	53.3	
60 and older	97	21.4	5	4.7	
Unit					< 0.001
DAST	5	1.1	15	14.0	
Hospital das Clínicas of UFMG	315	69.5	57	53.3	
Institute of Biological Sciences	40	8.8	4	3.7	
Veterinary School	11	2.4	17	5.9	
Other units	82	18.1	14	13.1	
Position					< 0.001
Nursing technician	468	10.9	444	10.6	
Laboratory technician	301	7.0	295	7.0	
Nursing assistant	222	5.1	197	4.7	
Physician	216	5.0	204	4.9	
Nurse	134	3.1	129	3.1	
Laboratory assistant	46	1.0	36	0.8	
Laboratory aide	34	0.7	32	0.7	
Agricultural assistant	24	0.5	19	0.4	
Chemistry technician	24	0.5	22	0.5	
Mechanical technician	12	0.2	14	0.3	
Agricultural technician	7	0.1	19	0.4	
Operational assistant	5	0.1	5	0.1	
Metallurgical technician	4	0	5	0.1	
Mining technician	4	0	5	0.1	
Veterinary and animal science assistant	1	0	1	0.0	
Food and dairy technician	0	0	4	0.1	

UFMG = Federal University of Minas Gerais; DAST = Departamento de Atenção à Saúde do Trabalhador (Department of Worker Health Care).

* Chi-square test.

Among employees losing the unhealthy work allowance, significant differences emerged for other evaluated characteristics (p < 0.001) (Table 4). The most common education level in this group was undergraduate degree (42.4%). This loss likely reflects unit transfers at HCUFMG. Whenever an employee moves units, the unhealthy work allowance is suspended. Employees must submit a new request for unhealthy work allowance when they begin work in a new unit. Such transfers are common at the hospital, which accounts for most staff in unhealthy roles. This also explains the rise in recipients holding an undergraduate degree (62.6%).

Secondary/technical education was the second most affected group: 35.1% lost the unhealthy work allowance and only 15.9% of them began receiving it. In 2021, UFMG had 4,208 TAE, of whom 2,934 (69.7%) held secondary/technical qualifications. At this level, nursing technicians, laboratory technicians and nursing assistants were the primary roles performing unhealthy activities. Therefore, the majority of these workers are exposed to biological hazards. Losses of unhealthy work allowance likely stem from the same factors identified for employees with undergraduate degrees, namely transfers between units within HCUFMG.

Only 13.2% of postgraduate employees lost the unhealthy work allowance, while 17.8% began receiving it. Health sector positions led both gains and losses, reflecting transfers between work units.

Employees aged 40 -59 years lost the allowance most often (62.9%) (Table 4). They comprise roughly 53.0% of TAE. Workers in that same age range also led among new recipients (53.3%). The 20-39 group had the lowest loss rate (15.7%) and ranked second among new recipients. Employees aged 60 years and older were least likely to begin receiving the unhealthy work allowance.

At UFMG, the HCUFMG remains the primary site for staff both losing and gaining unhealthy work allowance, as it houses most workers in unhealthy roles and sees frequent transfers between units (Table 4). At DAST, 1.1% of employees lost the allowance while 14.0% began receiving it in 2021, reflecting the expert’s new recognition of compensation for medical examiners. The School of Veterinary Medicine saw a notable change: in 2021, 2.4% of its staff lost eligibility and 15.9% started receiving compensation for occupational risk. This shift follows an increase in TAE at that unit from 33 in 2018 to 47 in 2021, of whom 11 (78.5%) now receive the allowance. In other UFMG units and sectors, 18.1% of employees lost compensation and 13.1% began receiving it.

## DISCUSSION

According to Rabinowitz & Rabinowitz,^4^ women represent 40% of physicians and 75% of the health workforce. The Pan American Health Organization (PAHO)^5^ reports that 86% of nurses in the Americas are female. These figures mirror the global gender distribution in this sector. Considering this, targeted women’s health initiatives for prevention and promotion become essential, given these workers’ critical role in maintaining effective health care delivery.

The COVID 19 pandemic affected men and women differently. Alkhouli et al.^6^ showed that male mortality from the virus is higher. Despite this, women have higher infection rates. The report “Mulheres no centro da luta contra a crise covid-19”^7^ states that women make up 70.0% of health workers worldwide. In Brazil, women constitute 85.0% of nursing staff, 45.6% of physicians and 85.0% of elder caregivers, exposing them to higher risk of viral infection. Beyond biological risk, notable issues include injuries from prolonged use of personal protective equipment, chemical exposure from disinfectants, increased workload and working hours, violence, discrimination, burnout, mental health disorders and poor conditions for health, hygiene and rest.

Baggenstoss et al.^8^ also reported that domestic violence during the pandemic was driven by several factors, including women’s confinement in their homes, which limited their social contact and ability to seek help, unemployment resulting from the economic effects of quarantine, which heightened domestic tensions, and increased alcohol consumption, among others.

Regarding male employees, according to Palotti & Freire,^9^ in 2014 men accounted for nearly 54% of federal public servants, while women accounted for 46%. That proportion varied by ministry. In some, male staff predominated, such as in the Ministry of Justice, the Ministry of Agriculture, Livestock and Food Supply, and the Ministry of Transport. In other ministries, women predominated, typically in the social portfolios such as the Ministry of Health, the Ministry of Social Security and the Ministry of Social Development and Fight Against Hunger. These patterns appear driven more by gender differences in university course choice and professional paths than by bias in career recruitment. However, women remain underrepresented in the elite careers of the Federal Executive Branch. The lower female presence partly explains their reduced participation in the most strategic commissioned posts, since those roles are typically filled by officers from these elite careers.^9^

According to Palotti & Freire,^9^ over the past two decades, rising recruitment into graduate level positions via public exams has underscored demand for more qualified staff, while hiring for mid and basic level roles has stabilized. Between 1997 and 2014, servers in mid-level and basic level positions fell from approximately 61.0% (332,057) to 40.0% (244,360) of all staff. Holders of higher education positions rose from 34.0% (182,303) to 48.0% (296,552). In the Federal Executive Branch, public exams for higher education positions climbed from 39.2 to 57.7% of all entries in that period, with 30.0% of those posts for university professors.

Health-related job categories received the highest number of unhealthy work allowances in both 2018 and 2021. Nursing was the leading category and saw the largest rise in recipients, followed by physicians. This growth may reflect expanded demand for these professionals, from new openings or expected retirements in these roles.

Silva et al.^10^ report that Federal Institutions of Higher Education (Instituições Federais de Ensino Superior, IFES) operate under specific legislation. References for TAE include Law No. 11.091/2005, which defines the Career Plan for Technical Administrative Positions in Education (Plano de Carreira dos Cargos Técnico Administrativos em Educação, PCCTAE); Decree No. 5.284/2006, which regulates the qualification incentive and classification by skill level for PCCTAE members; Decree No. 5.825/2006, which establishes guidelines for drafting the Development Plan for PCCTAE members; and Decree No. 7.232/2010, which grants IFES some autonomy in hiring new TAE. Thus, the career plan encourages federal university staff to pursue further studies and specialization, resulting in higher education levels.

Godoy^11^ notes that the law establishing the PCCTAE and the amendments introduced by Federal Law No. 11.784/2008 produced salary gains through staff qualification. A TAE employee holding a postgraduate specialization, master’s or doctoral degree thus becomes, in theory, best prepared for administrative-institutional challenges and, from a financial standpoint, more costly to the public treasury, with compensation potentially increased by incentives ranging from 10 to 75%.

According to the Painel Estatístico de Pessoal, the website providing data on federal civil servants, in 2018, 5,946 employees of the Ministry of Education retired. Of these, 41.0% were from the Southeast region, 93.0% retired voluntarily, 52.8% held higher education positions and 52.5% were women. In 2021, through May (the latest data available on the site at the time of consultation), 1,191 employees retired. As in 2018, the Southeast region (36.1%) was the most affected, where 91.1% retired voluntarily, 59.9% held higher education positions and 53.2% were women.

Ministry of Education staff retiring in those years had a different profile than federal civil servants overall. In 2021, so far, approximately 4,058 employees have retired. Of these, 92.5% retired voluntarily, 57.7% had secondary education and 52.7% were male. In 2018, that number was much higher, at 18,830 employees, of whom 93.6% retired voluntarily, 56.4% had secondary education and 51.4% were male.

Retirements rose significantly in 2019. That increase stems from changes to retirement rules introduced on November 12, 2019, by Constitutional Amendment No. 103. After that amendment, thousands of employees who had completed their service time requested to leave the public service, fearing they would be affected by its measures.

Workers aged 60 years and older received the fewest unhealthy work allowances. In 2019, retirements rose among employees who met the prerequisites (one being age), driven by concern over changes introduced by Constitutional Amendment No. 103, which revised retirement rules. After 2019, retirements fell sharply. This decline may relate to the COVID 19 pandemic, as economic impacts led employees to delay applying for retirement and to not meet the requirements needed to request the benefit.

## CONCLUSIONS

This study mapped the profile of UFMG employees performing unhealthy work activities. Across all periods, most were women (over 70%), aged 40 to 60 years, held an undergraduate degree, occupied health sector roles and worked in a hospital setting (over 80%).

The SIASS Concession Module for Occupational Allowances improved the granting process across federal bodies. It ensured compliance with existing legislation, enabled transparent computerized keeping, and accelerated operations by storing reports by unit and position. These benefits support its continued use and expansion to all federal public institutions.

There was a 26.8% reduction in employees, 30.0% in units, and 64.7% in OUs with recognized compensable unhealthy conditions. This change stems from various factors, notably the adoption of the computerized system. However, the decrease in allowance payments for employees exposed to physical and chemical hazards, which must be quantified under specific standards and laws to assess entitlement, resulted not from the SIASS module but from the lack of risk measurements due to procurement issues. Another factor is the decline in staff over the years, driven by labor outsourcing and the growth of workers affiliated with Empresa Brasileira de Serviços Hospitalares, a public company linked to the Ministry of Education, created to provide medical hospital and outpatient services within the Unified Health System (Sistema Único de Saúde, SUS), as well as support services for teaching, research and extension at federal higher education institutions and training in public health.

The remote management system standardized the allowance granting process, enabled remote access to information on work environments and activities, and supported audits and inspections, which reduced unhealthy work allowance expenses, especially in areas with biological hazards where exposure evaluations rely on qualitative methods.

Access to any information enables greater oversight and the taking of appropriate measures against irregularities. Thus, the SIASS module also serves to prevent infractions in granting this payment, ensuring appropriate use of public funds. However, it should also focus on intervening in unhealthy work environments to prevent illness and accidents and to support worker health promotion.

## References

[1] Brasil (1943). Aprova a consolidação das leis do trabalho.
Presidência da República.

[2] Brasil (1978). Aprova as normas regulamentadoras - NR - do capítulo V,
título II, da consolidação das leis do trabalho,
relativas a segurança e medicina do trabalho.

[3] Oliveira SG. (2011). Proteção jurídica à saúde do
trabalhador.

[4] Rabinowitz LG, Rabinowitz DG. (2021). Women on the frontline: a changed workforce and the fight against
COVID-19. Acad Med.

[5] Pan American Health Organization (2021). Reports from the region of the Americas.

[6] Alkhouli M, Nanjundappa A, Annie F, Bates MC, Bhatt DL. (2020). Sex differences in case fatality rate of COVID-19: insights from
a multinational registry. Mayo Clin Proc.

[7] Organização das Nações Unidas, ONU
mulheres Brasil (2020). Mulheres e meninas devem estar no centro dos esforços de resposta
à covid-19, apontam mulheres líderes.

[8] Baggenstoss GA, Li LP, Bordon LG. (2020). Violência contra mulheres e a pandemia do covid-19:
insuficiência de dados oficiais e de respostas do estado
brasileiro. Rev Dir Publico.

[9] Palotti P, Freire A. (2015). Servidores públicos federais: novos olhares e
perspectivas.

[10] Silva ML, Araújo ET, Dantas LMV. (2018). Perfil de competências dos servidores
técnico-administrativos em uma jovem universidade: novos perfis em
uma tradicional burocracia?. Interfaces Cient Direito.

[11] Godoy MT. (2014). Qualificação do servidor público:
implicações na gestão de pessoas na Universidade
Federal de Goiás.

